# Sociodemographic and clinical follow-up profile of transgender people accessing pre-exposure prophylaxis for the risk of HIV transmission in São Paulo, Brazil (2018-2021)

**DOI:** 10.1590/S2237-96222024v33e2024342.especial.en

**Published:** 2024-12-16

**Authors:** Marcos Morais Santos Silva, Denize Lotufo Estevam, Mateus Ettori Cardoso, Lucia Yasuko Izumi Nichiata

**Affiliations:** 1 Universidade de São Paulo, Escola de Enfermagem, São Paulo, SP, Brazil; 2 Centro de Referência e Treinamento DST/AIDS-SP, Coordenação Estadual de DST/AIDS de São Paulo, São Paulo, SP, Brazil; 3 Universidade de São Paulo, Escola de Enfermagem, São Paulo, SP, Brazil

**Keywords:** Profilaxis Pre-exposición, Personas Transgénero, Acceso a la Tecnología en Salud, VIH, Epidemiología Descriptiva, Pre-Exposure Prophylaxis, Transgender people, Access to Health Technology, HIV, Descriptive Epidemiology

## Abstract

**Objective:**

To describe the sociodemographic and clinical follow-up profile of the use of pre-exposure prophylaxis (PrEP) of HIV among transgender people receiving care at a reference health service for sexually transmitted infections and HIV/AIDS in São Paulo, the capital city of São Paulo state, between 2018 and 2021.

**Method:**

This was a descriptive study with an analysis of sociodemographic data, reasons for seeking PrEP , discontinuation of use and experiences of clinical follow-up. Descriptive statistics were used.

**Results:**

Among the 53 individuals, the majority were mixed-race (n= 25), transgender women (n= 48), heterosexual (n= 38) and had more than 11 years of study (n= 22). There was a decrease in follow-up visits (n= 14 to n= 3) after the second medical consultation.

**Conclusion:**

It is necessary to develop strategies to increase PrEP dispensing and continuation among transgender people, especially among Black people and those with lower level of education.

## Study contributions

**Main results:**

The majority of pre-exposure prophylaxis (PrEP) users were young, mixed-race, educated transgender women, reflecting a portion of the transgender population with access to healthcare. The discontinuation of clinical follow-up, especially after the second medical consultation, requires attention.

**Implications for services:**

Individualized strategies should be prioritized to improve the dispensing of PrEP and clinical follow-up for both transgender men and transgender women.

**Perspectives:**

Further research should focus on investigation of strategies to increase PrEP dispensing and clinical follow-up for transgender people, with an emphasis on transgender men, Black people and those with lower level of education.

## RESUMEN

**Objetivo:**

Describir el perfil sociodemográfico y el seguimiento del uso de la profilaxis preexposición (PrEP) para el VIH entre las personas transexuales atendidas en un servicio de salud de referencia para infecciones de transmisión sexual y VIH/sida en la ciudad de São Paulo entre 2018 y 2021.

**Método:**

Estudio descriptivo que analizó datos sociodemográficos, motivos de búsqueda de PrEP, interrupción del uso y experiencias de seguimiento clínico. Se utilizó estadística descriptiva.

**Resultados:**

Entre las 53 personas, la mayoría eran morenas (n=25), mujeres trans (n=48), heterosexuales (n=38) y con más de 11 años de escolaridad (n=22). Hubo una disminución de las visitas de seguimiento (de n=14 a n=3) a partir de la segunda consulta.

**Conclusión:**

Es necesario desarrollar estrategias para aumentar la dispensación y la continuidad del uso de la PPrE entre las personas trans, especialmente entre las personas de raza negra y con menor nivel de escolarización.

## INTRODUCTION

Pre-exposure prophylaxis (PrEP) of HIV involves the provision and use of a combination of antiretroviral drugs. This method has been proven effective in preventing HIV transmission. It has shown a reduction in infections in the United States, the United Kingdom, Australia, Kenya, Lesotho, Thailand and Vietnam.^1-3^

Despite the potential of PrEP to interrupt transmission and prevent new infections, there are disparities in access to this medication that need to be addressed to ensure equity in its use.^4^ There is growing concern about the barriers to access faced by transgender people, often resulting from negative and stigmatizing experiences within health services.^5-7^

An increase in HIV prevalence was observed in a systematic review, ranging from 18% in 2012 to 24% in 2021.^8^ This increase is not believed to be related to PrEP use itself, but rather that the provision of this procedure for transgender women has been limited. Health services are generally unprepared to meet the specific needs of the transgender population in an inclusive and sensitive manner. Barriers in accessing health services may have contributed to the increased HIV prevalence in this group. Such barriers remain one of the main challenges for the transgender population in the context of PrEP.^5-7^

Social marginalization and discrimination experienced by transgender people result in lower utilization of healthcare services.^9^ This situation is exacerbated by barriers such as lack of information about PrEP, bias in healthcare settings, and health care professionals’ concerns about drug interactions, which can discourage transgender people from seeking or continuing treatment.^10^ The intersectionality of factors such as race/skin color, socioeconomic status, and contexts of vulnerability, including geographic location – such as peripheral areas – can worsen access to preventive measures, creating significant disparities in access to PrEP among transgender people.^10^

Longitudinal studies, for example, underrepresent transgender women.^11^ In Brazil, there is an insufficient or inadequate representation of transgender people in studies on oral PrEP. Analyses of such studies are often grouped together with men who have sex with men.^12-14^ In developed countries, the majority of people who use prophylaxis are men who have sex with men, of White/race skin color, and with a high level of education.^5-17^ This profile is similar to that presented in Brazil,^19^ which may reflect those who effectively access healthcare services, although there is an unknown population that is unable to access such services. In the case of transgender people, little is known about the profile of those who access PrEP.

Therefore, studies evaluating transgender people who do not have other sexual orientations are therefore necessary. These analyses would enable a better understanding of the profile of access to prophylaxis among transgender people. This study aimed to describe the sociodemographic profile and clinical follow-up of the use of PrEP of HIV among transgender people receiving care at a reference health service for sexually transmitted infections and HIV/AIDS in São Paulo, the capital city of São Paulo state, between 2018 and 2021.

## METHOD

### Study design and setting

This was a descriptive study that included adult transgender men and women who received PrEP at the STD/AIDS Reference and Training Center in São Paulo.

### Setting

The healthcare service is a state-level reference for the diagnosis and treatment of sexually transmitted infections. It was one of the first HIV reference and training centers in Brazil, established in 1988, and includes a testing and counseling center, emergency room, clinical analysis laboratory, day and 24-hour hospitalization, HIV/AIDS, viral hepatitis and transvestite and transgender outpatient clinics. It provides care for approximately 7,000 users per month. It is staffed by multidisciplinary teams comprised of nurses, infectious disease doctors and specialists, pharmacists, psychologists, biologists, among other professionals.

### Participants

Transgender men and women aged 18 years or older who self-identified as such and received at least one combination of PrEP medications between January 2018 and June 2021 were included. Individuals who received PrEP and were transferred to another healthcare service were excluded, due to the unavailability of data following the transfer. This period was prioritized to ensure uniformity in data collection forms, which were modified after 2021. Clinical follow-up was considered up to the third medical consultation in order to minimize loss to follow-up.

### Variables

The variables included in this study were: age (in years: 18-24, 25-29, 30-34, > 35), country of origin, gender identity (transgender man, transgender woman), sexual orientation (bisexual, heterosexual, homosexual), race/skin color (White, Indigenous, mixed-race, Black), homeless (no, yes), schooling (in years: 1-3, 4-7, 8-11, > 11), reason for attending the healthcare service (general information/care, PEP, PrEP), reason for seeking PrEP (referred by a healthcare professional, informed by a non-governmental organization, awareness through printed communication or internet), previous PrEP (self-initiative, did not start, participated in a research project), sex with an HIV-positive partner (no, yes, do not know), sex in exchange for money (no, yes), use of injectables (no, yes), needle sharing for steroid use (no, yes), history of kidney problems (no, don’t know, yes), loss to follow-up after medical consultations (no; yes, but returned; yes and did not return); prescriber (nurse, doctor), frequency of condom use (never, less than half the time, half the time, more than half the time, always), alcohol use (no, yes), substance use (no, yes: nitrite, cocaine, crack, marijuana, party drugs, male sexual stimulant, solvent), adverse effects from PrEP (no, yes: diarrhea, flatulence, nausea, vomiting, abdominal pain, other adverse effects), reason for stopping PrEP (Lack of medication, adverse effects, forgetfulness, absence from home, other), hepatitis B vaccination (one dose, two doses, complete schedule, referred for vaccination).

The variables were categorized into sociodemographic factors, reasons for seeking PrEP , PrEP eligibility criteria , HIV infection risk assessment, potential PrEP exclusion criteria, adverse effects, PrEP adherence , laboratory results and final procedures and were collected at first visit and at two subsequent times (Table 1).

**Box 1:** Variables collected during clinical follow-up of pre-exposure prophylaxis (PrEP), São Paulo, Brazil, 2018-2021

Initial appointment
Sociodemographic characteristics and PrEP use
Reason for attending the Reference and Training Center
Reason for seeking PrEP
**Second and third follow-ups**
Loss to follow-up
Prescriber
Frequency of condom use
Alcohol use
Substance use
PrEP adverse effects
Reason for discontinuing PrEP
Hepatitis B vaccination

### Data source and measurement

Data were obtained from the Medication Logistic Control System (*Sistema de Controle Logístico de Medicamentos* - SICLOM), available at https://siclom.aids.gov.br . These data include: (i) first consultation form for PrEP (user’s first day at the health service and PrEP initiation); (ii) the follow-up form 30 days after prophylaxis initiation; and (iii) the PrEP clinical follow-up form (completed every 30, 60, 90 or 120 days, depending on the number of pills dispensed by the healthcare professional).

Discontinuation was defined as when the individual did not return the healthcare service after exceeding 40% the days covered by the medication provided during the last visit, as recommended by the Ministry of Health.^18^

### Data analysis 

The anonymized data were compiled in Microsoft Excel and analyzed descriptively using the R Studio, version 4.1.1.

### Ethical aspects 

The study was approved by the Research Ethics Committee (REC) of the Escola de Enfermagem da Universidade de São Paulo, opinion No. 6,128,586 of 06/19/2023, certificate of submission for ethical appraisal (CAAE) 69454123.8.0000.5392, and by the REC of the DST AIDS Reference and Training Center in São Paulo, Opinion No. 6,267.75 of 08/29/2023, CAAE 69454123.8.3001.5375.

### RESULTS

A total of 53 transgender people were included in the study (Table 1). The majority of people were Brazilian (n=51), transgender women (n=48), heterosexual (n=38), mixed-race (n=25), lived in their own or rented housing (n=48) and had more than 11 years of study (n=22).

The primary motivation for seeking PrEP was the individual’s own desire to access it (n=51), followed by awareness raised through printed communication or online media (n=31). Sex in exchange for money was reported by 43 participants. Injectable drug use and sharing of equipment were recorded in 1 and 2 cases, respectively (Table 1). A decrease in follow-up attendance and in the number of those who did not return after the initial loss to follow-up (from n=14 at the first medical consultation to n=3 at the third medical consultation). Most prescriptions were written by doctors during the first consultation (n=36), the second (n=24) and the third (n=16) (Table 2).

**Table 1:** Sociodemographic characteristics and reasons for seeking and criteria for pre- exposure prophylaxis (PrEP) use by transgender people, São Paulo, Brazil, 2018-2021 (n=53)

Variable	n
Age (years)	
18-24	14
25-29	15
30-34	10
≥35	14
**Country of origin**	
Brazil	51
Italy	1
Venezuela	1
**Gender identity**	
transgender man	5
Transgender woman	48
**Sexual orientation**	
Bisexual	7
Heterosexual	38
Homosexual	8
**Race/skin color**	
White	18
Indigenous	3
Mixed-race	25
Black	7
**Homeless**	
No	48
Yes	3
**Schooling (years)**	
1-3	1
4-7	11
8-11	19
>11	22
**Reason for attending health service**	
General information/service	1
PEP	1
PrEP	51
**Reason for seeking PrEP**	
Referred by healthcare professional	19
Guided by non-governmental organization	3
Awareness through printed communication or internet	31
**Previous PrEP use** ^a^	
Self-initiative	5
Did not start	25
Participated in a research project	22
**Sex with HIV-positive partner**	
No	25
Yes	5
Do not know	23
**Exchange of sex for money**	
No	10
Yes	43
**Use of Injectables** ^a^	
No	51
Yes	1
**Sharing syringes for the use of anabolic steroids** ^a^	
No	50
Yes	2
**History of kidney problems** ^a^	
No	46
Do not know	3
Yes	2

a) Missing data due to incomplete records by the healthcare professional.

**Table 2:** Characteristics of pre-exposure prophylaxis (PrEP) according to follow-up consultations, São Paulo, Brazil, 2018-2021 (n=53)

Variables	First	Second	Third
**Loss to follow-up after consultations**			
No	25	17	7
Yes, but returned	9	5	7
Yes and did not return	14	6	3
Prescriber			
Nurse	17	10	6
Doctor	36	24	16
**Frequency of condom use**			
Never	3	1	1
Less than half of the time	5	0	2
Half of the time	4	0	1
More than half of the time	24	0	8
Always	17	0	7
**Alcohol use** ^a^			
No	1	1	14
Yes	0	0	6
**Substance use**			
No	16	1	14
Yes	37	0	6
Nitrite	2		0
Cocaine	17		2
Crack	2		0
Marihuana	26		1
Party drugs	11		3
Sexual stimulant	4		2
Solvent	4		0
**Adverse effects of PrEP**			
No	-	25	19
Yes	-	8	1
Diarrhea	-	5	0
Flatulence	-	2	0
Nausea	-	1	0
Vomiting	-	1	0
Abdominal pain	-	2	1
Other adverse effects	-	2	0
**Reason for discontinuing PrEP ^a^**			
Lack of medication	-	1	3
Adverse effect	-	1	0
Forgetfulness	-	3	0
Absence from home	-	0	0
Other	-	2	0
**Hepatitis B vaccination** ^a^			
One dose	1	5	3
Two doses	0	3	4
Complete schedule	29	20	12
Forwarded for vaccination	15	0	0

a) Missing data due to incomplete by healthcare professional.

A large proportion reported using condoms during sexual intercourse most of the time (n=24 at the first consultation) or always (n=17 at the first consultation). Many responses regarding alcohol use were missing. Most participants reported using illicit drugs during the first evaluation (n=37); at the third evaluation, there were (n=6). The most frequently used substances were cocaine, marijuana and party drugs (Table 2).

Most participants did not report adverse effects from PrEP use (n=25) at the first consultation (n=19 at the second). Diarrhea was the most commonly cited issue among the reported adverse effects. The main reasons for discontinuing PrEP were forgetting to take antiretroviral drugs (n=3 at the first consultation) and running out of medication. The majority of participants completed the hepatitis B vaccination schedule (n=29) at the first consultation, and n=20 at the second (Table 2).

## DISCUSSION

The majority of transgender people who received PrEP at a reference health service in São Paulo were young adults, transgender women, of mixed-race/skin color, and had complete high school. These data align with the profile identified by the Ministry of Health, which shows a predominance of transgender women with higher level of education among this population.^18^

The study population was limited to people who have access to health services and thus the possibility of obtaining medication for HIV prevention. This profile potentially excludes the experiences of Black transgender women and men who have lower level of education, as well as their access to PrEP.

Socially vulnerable transgender women face greater difficulty accessing health services^7^ and PrEP.^19^ Improving access to education and income for transgender people, as well as the organization of health services, is essential, given these barriers and the need to overcome them.^20^

Illicit drug use was frequently reported among transgender people using PrEP, particularly cocaine, at the start of prophylaxis. Since people using PrEP will be followed over time, this represents an opportunity for healthcare professionals to address broader issues, such as harm reduction, which is necessary for people using PrEP.^21^

This study highlighted the importance of health services in preventing hepatitis B, underscoring the need to check and start vaccination during the first PrEP consultation. The follow-up of people on PrEP may represent an opportunity to update their vaccination schedule. In Canada, 46% of cisgender men and women and transgender women with hepatitis B did not receive adequate monitoring during their PrEP follow-up between 2018 and 2019.^16^ Increasing awareness and education about hepatitis B among professionals who prescribe PrEP is therefore a priority.^22^ This approach is particularly important when considering the high prevalence of hepatitis B among transgender people, requiring inclusive and accessible strategies for this population.^23^

In this study, few individuals reported adverse effects, most of which were mild, similar to the findings of a systematic review.^24,25^ These effects should not pose barriers to the use of PrEP, minimizing health professionals’ concerns about adverse effects from antiretroviral drugs. in PrEP prescription.^5^ The occurrence of adverse effects should be monitored by these professionals, as well as efforts to remove barriers to PrEP access.

Discontinuation of PrEP use due to forgetfulness was observed in this study. In other therapeutic regimens that require regular medication intake, such as diabetes treatment and HIV infection, the use of digital technologies, such as cell phone alerts and reminders in electronic calendars, can help with medication adherence.^26^

The use of PrEP is primarily determined by the user through an evaluating process with the health professional regarding the risks of transmission. There is no adherence in the same sense as in infection treatment. Given the follow-up through health services, this is an important factor to be analyzed, regarding PrEP continuation, due to its relevance for HIV prevention. In this study, many transgender people discontinued PrEP follow-up as early as the second consultation. A study involving 294 transgender people in the United States enrolled between October 2017 and May 2018 revealed that 49% discontinued use after one year.^11^

The reasons for seeking PrEP show the importance of healthcare professionals and non-governmental organizations in facilitating access to prophylaxis. It is necessary to expand partnerships between health services and various social organizations to establish a collaborative network that promotes prevention and create more welcoming and judgment-free environments encouraging individuals to seek and continue follow-up care.^27^ Education and awareness campaigns on PrEP can also increase knowledge and acceptance of PrEP as an effective HIV prevention method. The study also showed the influence of social media in disseminating information about seeking PrEP. This is a resource that can be incorporated by health services, aligning with what is called a digital presence.^28^

This study has limitations that should be considered when interpreting the results. First, the sample size was small, with 53 transgender people followed throughout the study period. This may limit the generalization of the results to other transgender populations in different contexts. The study used secondary data from service records, which may have introduced biases due to potential incompleteness or inconsistency in the information recorded. Another limitation is the loss to follow-up observed during clinical follow-up, especially after the second visit. This loss may have influenced the findings, as the analysis considered the first three follow-up visits, which limited a comprehensive assessment of long-term PrEP continuity.

In conclusion, the majority of transgender people using PrEP at a reference health service in São Paulo were young, mixed-race transgender women with higher level of education. High discontinuation rates in clinical follow-up after the second consultation were observed, suggesting the need for actions to increase PrEP continuity. Given this narrow window of opportunity, strategic prevention actions aimed at this population, such as hepatitis B vaccination, should be prioritized during the initial consultations.
