# Supplementary material for: Sociodemographic and clinical follow-up profile of transgender people accessing pre-exposure prophylaxis for the risk of HIV transmission in São Paulo, Brazil (2018-2021)
Source: Epidemiol Serv Saude. 2024 Dec 16;33(spe1):e2024342. doi: 10.1590/S2237-96222024v33e2024342.especial.en (PMC11654812; doi:10.1590/S2237-96222024v33e2024342.especial.en)
Supplement: Translation

# Translation: Perfil sociodemográfico e de acompanhamento clínico de pessoas trans que acessam a profilaxia pré-exposição ao risco de transmissão do HIV em São Paulo (2018-2021)

**DOI:** 10.1590/S2237-96222024v33e2024342.especial.pt

**Authors:** Marcos Morais Santos Silva, Denize Lotufo Estevam, Mateus Ettori Cardoso, Lucia Yasuko Izumi Nichiata

**Affiliations:** 1 Universidade de São Paulo, Escola de Enfermagem, São Paulo, SP, Brasil; 2 Centro de Referência e Treinamento DST/AIDS-SP, Coordenação Estadual de DST/AIDS de São Paulo, São Paulo, SP, Brasil; 3 Universidade de São Paulo, Escola de Enfermagem, São Paulo, SP, Brasil

**Keywords:** Profilaxia Pré-exposição, Pessoas Transgênero, Acesso à Tecnologia em Saúde, HIV, Epidemiologia Descritiva

## Abstract

**Objetivo:**

Descrever o perfil sociodemográfico e de acompanhamento do uso da profilaxia pré-exposição (PrEP) ao HIV entre pessoas trans atendidas em um serviço de saúde referência em infecções sexualmente transmissíveis e HIV/aids no município de São Paulo entre 2018 e 2021.

**Método:**

Estudo descritivo com análise de dados sociodemográficos, motivos de procura da PrEP, descontinuidade do uso e experiências de acompanhamento clínico. Empregou-se estatística descritiva.

**Resultados:**

Entre as 53 pessoas, a maioria era parda (n=25), mulheres trans (n=48), heterossexual (n=38) e com mais de 11 anos de estudos (n=22). Houve uma diminuição nas visitas de acompanhamento (n=14 para n=3) a partir da segunda consulta.

**Conclusão:**

É necessário desenvolver estratégias para aumentar a dispensação e continuidade do uso da PrEP entre pessoas trans, principalmente entre pretas e com menor escolaridade.

## Contribuições do estudo

**Principais resultados:**

A maioria dos usuários da profilaxia pré-exposição (PrEP) era mulher trans, jovem, parda e escolarizada, refletindo uma parcela da população trans com acesso à saúde. A descontinuidade no acompanhamento clínico, especialmente após a segunda consulta, requer atenção.

**Implicações para os serviços:**

Estratégias individualizadas devem ser priorizadas para melhorar a dispensação da PrEP e o acompanhamento clínico de homens e mulheres trans.

**Perspectivas:**

Pesquisas futuras deveriam priorizar investigação de estratégias para aumentar a dispensação e o acompanhamento clínico da PrEP para pessoas trans, com destaque a homens trans, pessoas negras e com menor escolaridade.

## INTRODUÇÃO

A profilaxia pré-exposição de HIV (PrEP) consiste na oferta e no uso de combinação de antirretrovirais. Esse método já está comprovado ser eficaz na prevenção da transmissão do HIV. Ele demonstrou uma redução da infecção nos Estados Unidos, no Reino Unido, na Austrália, no Quênia, em Lesoto, na Tailândia e no Vietnã.^1-3^

Apesar do potencial da PrEP para interromper a transmissão e evitar novas infecções, existem disparidades no acesso a esse medicamento que precisam ser abordadas para garantir a equidade em seu uso.^4^ Há preocupação crescente com as barreiras de acesso enfrentadas por pessoas trans, muitas vezes decorrentes de experiências negativas e estigmatizantes nos serviços de saúde.^5-7^

Aumento na prevalência do HIV foi observado em uma revisão sistemática, que passou de 18% em 2012 para 24% em 2021^8^.Discute-se que esse aumento não está relacionado ao uso da PrEP em si, mas que a oferta desse procedimento para mulheres trans tem sido limitada. Os serviços de saúde em geral não estão preparados para atender, de forma inclusiva e sensível, às necessidades específicas da população transgênero. As barreiras no acesso aos serviços de saúde podem ter contribuído para o aumento da prevalência do HIV nesse grupo. Tais barreiras são um dos principais desafios para a população trans no contexto da PrEP.^5-7^

A marginalização social e a discriminação que pessoas trans frequentemente enfrentam resultam em menor utilização dos serviços de saúde.^9^ Esse cenário é exacerbado por barreiras como a falta de informação sobre a PrEP, os preconceitos no atendimento de saúde e a preocupação dos profissionais de saúde com interações medicamentosas, que podem desencorajar pessoas trans a buscar ou continuar o tratamento.^10^ A interseccionalidade de condicionantes como raça/cor da pele, condição socioeconômico e contextos de vulnerabilidade, inclusive como localização geográfica,– a exemplo das regiões periféricas – , pode agravar o acesso às medidas preventivas, criando disparidades significativas no acesso à PrEP entre pessoas trans.^10^

Estudos longitudinais, por exemplo, subrepresentam mulheres trans.^11^ No Brasil, há uma representação insuficiente ou inadequada de pessoas trans em estudos da PrEP oral. As análises de tais estudos frequentemente são agrupadas junto aos homens que fazem sexo com homens.^12-14^ Em países desenvolvidos, a maioria das pessoas que fazem uso da profilaxia é composta por homens que fazem sexo com homens, brancos e com alto nível de escolaridade.^15-17^ Esse é um perfil semelhante ao apresentado no Brasil,^19^ que pode refletir aqueles que efetivamente acessam os serviços de saúde, embora exista contingente desconhecido que sequer consegue acessar tais serviços. No caso de pessoas trans, pouco se sabe sobre o perfil das que acessam a PrEP.

Estudos que avaliem pessoas trans não agregadas a outras orientações sexuais mostram-se, portanto, necessários. Tais análises possibilitariam entender o perfil de acesso à profilaxia entre as pessoas trans. Este estudo objetivou descrever o perfil sociodemográfico e o acompanhamento clínico do uso da PrEP ao HIV entre pessoas trans atendidas em um serviço de saúde referência em infecções sexualmente transmissíveis e HIV/AIDS no município de São Paulo, entre 2018 e 2021.

## MÉTODO

### Delineamento e contexto

Trata-se de um estudo descritivo que incluiu homens e mulheres trans adultos que receberam PrEP no Centro de Referência e Treinamento DST AIDS/SP.

### Contexto 

O serviço de saúde é referência estadual para o diagnóstico e tratamento de infecções sexualmente transmissíveis. Foi um dos primeiros centros de referência e treinamento em HIV no Brasil, criado em 1988 e conta com centro de testagem e aconselhamento, pronto atendimento, laboratório de análises clínicas, internação diurna e 24 horas, ambulatórios de HIV/aids, hepatites virais e de travestis e transexuais. Mensalmente atende cerca de 7 mil usuários. Conta com equipes multidisciplinares compostas por enfermeiros, médicos infectologistas e especialistas, farmacêuticos, psicólogos, biólogos, entre outros profissionais. 

### Participantes

Foram incluídos homens e mulheres trans com 18 anos ou mais que assim se identificaram e que receberam ao menos uma combinação de medicamentos PrEP entre janeiro de 2018 a junho de 2021. Foram excluídas pessoas que receberam PrEP e foram transferidas para outro serviço de saúde, devido a indisponibilidade dos dados após a transferência. Tal período foi priorizado para uniformidade nas fichas de coleta, que sofreu modificação após 2021. Foi considerado de acompanhamento clínico até a terceira consulta a fim de minimizar perdas de seguimento. 

### Variáveis

Foram incluídas como variáveis desse estudo: idade (em anos: 18-24, 25-29, 30-34, > 35), País de origem , identidade de gênero (homem trans, mulher trans), orientação sexual (bissexual, heterossexual, homossexual), raça/cor da pele (branca, indígena, parda, preta), situação de rua (não, sim), escolaridade (em anos: 1-3, 4-7, 8-11, >11), motivo de chegada ao serviço de saúde (informação/atendimento em geral, PEP, PrEP), motivo de busca pela PrEP (encaminhado por profissional de saúde, orientado por organização não governamental, sensibilizado por comunicação impressa ou internet), PrEP anterior (iniciativa própria, não iniciou, participou de projeto de pesquisa), sexo com parceiro HIV (não, sim, não sabe), troca sexo por dinheiro (não, sim), uso de injetáveis (não, sim), compartilhamento de seringas para uso de anabolizantes (não, sim), histórico de problema renal (não, não sabe, sim), perda de seguimento após consultas (não; sim, mas retornou; sim e não retornou); prescritor (enfermeiro, médico), frequência de uso do preservativo (nenhuma vez, menos da metade das vezes, metade das vezes, mais da metade das vezes, todas as vezes), uso de álcool (não, sim), substância (não, sim: nitrito, cocaína, crack, maconha, drogas de festas, estimulante de ereção, solvente), efeitos adversos por PrEP (não, sim: diarreia, flatulência, náuseas, vômitos, dor abdominal, outros efeitos adversos), motivo pelo qual deixou de tomar a PrEP (Falta de medicamento, efeito adverso, esquecimento, ausência do domicílio, outro), vacinação contra hepatite B (uma dose, duas doses, esquema completo, encaminhado para vacinação).

As variáveis de foram organizadas em sociodemográficas, razões para buscar a PrEP, critérios de elegibilidade da PrEP, avaliação de riscos de infecção pelo HIV, possíveis critérios de exclusão da PrEP, efeitos adversos, adesão à PrEP, resultados laboratoriais e condutas finais e foram coletadas no primeiro atendimento e em dois momentos subsequentes (Quadro 1).

**Quadro 1:** Variáveis coletadas no acompanhamento clínico da profilaxia pré-exposição (PrEP), São Paulo, 2018-2021

Primeiro atendimento
Características sociodemográficas e uso da PrEP
Motivo de chegada no Centro de Referência e Treinamento
Motivo de busca pela PrEP
**Segundo e terceiro retornos**
Perda de seguimento
Prescritor
Frequência de uso do preservativo
Uso de álcool
Uso de substâncias
Efeitos adversos da PrEP
Motivo de abandono da PrEP
Vacinação contra hepatite B

### Fonte de dados e mensuração

Os dados foram obtidos do Sistema de Controle Logístico de Medicamentos (Siclom), disponível em https://siclom.aids.gov.br. Esses dados incluem: (i) ficha do primeiro atendimento para PrEP (primeiro dia do usuário no serviço de saúde e início da PrEP); (ii) ficha do retorno de 30 dias após o início da profilaxia; e (iii) ficha de acompanhamento clínico da PrEP (preenchida a cada 30, 60, 90 ou 120 dias, a depender da quantidade de comprimidos dispensados pelo profissional de saúde). 

Considerou-se descontinuidade quando o indivíduo não compareceu ao serviço de saúde depois de exceder em 40% o período dos dias de fornecimento do medicamento estipulado na última visita, conforme preconizado pelo Ministério da Saúde.^18^

### Análise dos dados

Os dados anonimizadas foram compilados em Microsoft Excel e analisado descritivamente utilizando-se o programa R Studio, versão 4.1.1. 

### Aspectos éticos

O estudo foi aprovado pelo Comitê de Ética e Pesquisa (CEP) da Escola de Enfermagem da Universidade de São Paulo por meio do parecer 6.128.586 de em 19/06/2023, certificado de apresentação de apreciação ética (CAAE) 69454123.8.0000.5392, e pelo CEP do Centro de Referência e Treinamento DST AIDS/SP conforme o Parecer 6.267.75 de 29/08/2023, CAAE 69454123.8.3001.5375.

## RESULTADOS

Foram incluídas 53 pessoas trans (Tabela 1). A maioria das pessoas era brasileira (n=51), mulher trans (n=48), heterossexual (n=38), parda (n=25), residiam em moradia própria ou alugada (n=48) e frequentaram escola por mais de 11 anos (n=22). 

A principal motivação para a busca da PrEP foi o próprio desejo de acessá-la (n=51), seguido por sensibilização através de meios de comunicação impressos ou pela internet (n=31). A troca de sexo por dinheiro foi relatada por 43 participantes. O uso de drogas injetáveis e o compartilhamento de instrumentos foi registrado em 1 e 2 casos, respectivamente (Tabela 1). Nota-se a diminuição de comparecimento e dos que não retornaram após a perda inicial de seguimento (de n=14 na primeira consulta para n=3 na terceira consulta). A maioria das prescrições foi feita por médicos na primeira consulta (n=36), na segunda (n=24) e na terceira (n=16) (Tabela 2).

**Tabela 1:** Caracterização sociodemográfica e motivos da busca e critérios de uso da profilaxia pré-exposição (PrEP) por pessoas trans, São Paulo, 2018-2021 (n=53)

Variável	n
Idade (anos)	
18-24	14
25-29	15
30-34	10
≥35	14
**País de origem**	
Brasil	51
Itália	1
Venezuela	1
**Identidade de gênero**	
Homem trans	5
Mulher trans	48
**Orientação sexual**	
Bissexual	7
Heterossexual	38
Homossexual	8
**Raça/cor da pele**	
Branca	18
Indígena	3
Parda	25
Preta	7
**Situação de rua** ^a^	
Não	48
Sim	3
**Escolaridade (anos)**	
1-3	1
4-7	11
8-11	19
>11	22
**Motivo de chegada ao serviço de saúde**	
Informação/atendimento em geral	1
PEP	1
PrEP	51
**Motivo de busca pela PrEP**	
Encaminhado por profissional de saúde	19
Orientado por organização não governamental	3
Sensibilizado por comunicação impressa ou internet	31
**PrEP anterior** ^a^	
Iniciativa própria	5
Não iniciou	25
Participou de projeto de pesquisa	22
**Sexo com parceiro HIV**	
Não	25
Sim	5
Não sabe	23
**Troca sexo por dinheiro**	
Não	10
Sim	43
**Uso de injetáveis** ^a^	
Não	51
Sim	1
**Compartilhamento de seringas para uso de anabolizantes** ^a^	
Não	50
Sim	2
**Histórico de problema renal** ^a^	
Não	46
Não sabe	3
Sim	2

a) Dado faltante devido ao não preenchimento pelo profissional de saúde.

Grande parte reportou uso de preservativo nas relações sexuais na maioria das vezes (n=24 na primeira consulta) ou em todas as vezes (n=17 na primeira consulta). Muitas respostas não foram informadas sobre o uso de álcool. A maioria informou uso de drogas ilícitas na primeira avaliação (n=37); na terceira avaliação, foram (n=6). As substâncias que mais se destacaram foram a cocaína, a maconha e as drogas de festas (Tabela 2).

**Tabela 2:** Características da profilaxia pré-exposição (PrEP) de acordo com as consultas de avaliação, São Paulo, 2018-2021 (n=53)

Variáveis	Primeira	Segunda	Terceira
**Perda de seguimento após consultas**			
Não	25	17	7
Sim, mas retornou	9	5	7
Sim e não retornou	14	6	3
**Prescritor**			
Enfermeiro	17	10	6
Médico	36	24	16
**Frequência de uso do preservativo**			
Nenhuma vez	3	1	1
Menos da metade das vezes	5	0	2
Metade das vezes	4	0	1
Mais da metade das vezes	24	0	8
Todas as vezes	17	0	7
**Uso de** **á** **lcool** ^a^			
Não	1	1	14
Sim	0	0	6
**Substâncias**			
Não	16	1	14
Sim	37	0	6
Nitrito	2		0
Cocaína	17		2
Crack	2		0
Maconha	26		1
Drogas de festas	11		3
Estimulante de ereção	4		2
Solvente	4		0
**Efeitos adversos por PrEP**			
Não	-	25	19
Sim	-	8	1
Diarreia	-	5	0
Flatulência	-	2	0
Náuseas	-	1	0
Vômitos	-	1	0
Dor abdominal	-	2	1
Outros efeitos adversos	-	2	0
**Motivo pelo qual deixou de tomar a PrEP** ^a^			
Falta de medicamento	-	1	3
Efeito adverso	-	1	0
Esquecimento	-	3	0
Ausência do domicílio	-	0	0
Outro	-	2	0
**Vacinação contra hepatite B** ^a^			
Uma dose	1	5	3
Duas doses	0	3	4
Esquema completo	29	20	12
Encaminhado para vacinação	15	0	0

a) Dado faltante devido ao não preenchimento pelo profissional de saúde.

A maioria não relatou efeitos adversos com o uso da PrEP (n=25) na primeira consulta, (n=19 na segunda). A diarreia foi o problema mais citado entre os efeitos adversos relatados. Os principais motivos para a interrupção da PrEP foram o esquecimento na tomada dos antirretrovirais (n=3 na primeira consulta) e o término do medicamento. A maioria completou o esquema de vacinação para hepatite B (n=29) na primeira consulta, n=20 na segunda (Tabela 2).

## DISCUSSÃO

A maioria das pessoas trans que receberam PrEP em serviço de referência paulistano era formada por adultos jovens, mulheres trans, pardas e que estudaram até o ensino médio. Esses dados se alinham com perfil diagnosticado pelo Ministério da Saúde, de predominância de mulheres trans com maior escolaridade dentre essa população.^18^

A população de estudo se restringe a pessoas que conseguiram acesso a serviço de saúde, tendo a possibilidade de obter medicamento para prevenção do HIV. Tal perfil potencialmente exclui a experiência de mulheres e homens trans menos escolarizadas e pretas, bem como o acesso dessa população a PrEP.

Mulheres trans que vivem situação de vulnerabilidade social têm mais dificuldade de acesso aos serviços de saúde^7^ e à PrEP.^19^ Melhorar o acesso das pessoas trans à educação e renda, assim como na organização dos serviços de saúde se mostra necessário, considerando essas barreiras e formas de superá-las.^20^

O uso de drogas ilícitas entre as pessoas trans em uso da PrEP foi frequente no início da profilaxia, em especial de cocaína. Como as pessoas em uso da PrEP serão acompanhadas ao longo do tempo, isso representa uma oportunidade para que os profissionais de saúde trabalhem com questões mais amplas, a exemplo da redução de danos, necessário para pessoas que fazem uso da PrEP.^21^

Este estudo evidenciou a importância dos serviços de saúde na prevenção da hepatite B, destacando a necessidade de checar e iniciar a vacinação já na primeira consulta de PrEP. O acompanhamento de pessoas em PrEP pode representar oportunidade de atualização do calendário vacinal. No Canadá, 46% de homens e mulheres cis e mulheres trans com hepatite B não receberam monitoramento adequado durante o acompanhamento da PrEP entre 2018 e 2019. ^16^ Aumentar a conscientização e a educação sobre a hepatite B entre os profissionais que prescrevem a PrEP se mostra prioritário.^22^ Tal conduta se mostra mais relevante ao considerar a elevada prevalência de hepatite B em pessoas trans, por meio de estratégias inclusivas e acessíveis para essa população.^23^

Neste estudo, poucas pessoas apresentaram efeitos adversos, na maioria leves, semelhante aos achados de uma revisão sistemática.^24,25^ Tais efeitos não devem ser barreiras ao uso da PrEP, minimizando preocupação de profissionais da saúde sobre efeitos adversos por antirretrovirais na indicação da PrEP.^5^ Manifestação de efeitos adversos deve ser observada por esses profissionais, da mesma forma que a remoção assim como a remoção de barreiras no acesso à PrEP.

A descontinuidade do uso da PrEP decorrente do esquecimento foi observada no presente estudo. Em outros regimes terapêuticos que exigem a ingestão regular de medicamentos, como no tratamento de diabetes e na infecção pelo HIV, o uso de tecnologias digitais, como avisos no celular e lembretes em agendas eletrônicas, podem auxiliar na adesão a terapia medicamentosa.^26^

Por princípio, o uso de PrEP é determinado pelo usuário no processo de avaliação com o profissional de saúde em relação aos riscos de transmissão. Não há exatamente a adesão no mesmo sentido de tratamento da infecção. Considerando o acompanhamento nos serviços de saúde, esse é um dado importante a ser analisado, no sentido da manutenção do seguimento da PrEP, devido sua relevância na prevenção do HIV. Neste estudo, muitas pessoas trans deixaram de realizar o acompanhamento da PrEP e descontinuaram já na segunda consulta. Uma pesquisa com 294 pessoas trans nos Estados Unidos inscritas entre outubro de 2017 e maio de 2018, revelou que 49% descontinuaram o uso após um ano.^11^

Os motivos da busca pela PrEP mostram a importância dos profissionais de saúde e de organizações não governamentais para facilitar o acesso à profilaxia. É preciso ampliar as parcerias entre os serviços de saúde e equipamentos sociais diversos para instituir uma rede colaborativa para incentivo à prevenção e na promoção de ambientes mais acolhedores e sem julgamentos, que encorajam indivíduos a buscar e continuar o acompanhamento.^27^ Campanhas de educação e sensibilização sobre a PrEP também podem aumentar o conhecimento e a aceitação da PrEP como método eficaz de prevenção do HIV. O estudo inclusive mostrou a influência das redes sociais na divulgação de informações para a procura da PrEP. Esse é um recurso que pode ser incorporado pelos serviços de saúde, inserido no que é chamado presença digital.^28^

Este estudo apresenta limitações que devem ser consideradas ao interpretar os resultados. Primeiramente, o tamanho da amostra foi pequeno, com 53 pessoas trans acompanhadas ao longo do período de estudo. Isso pode limitar a generalização dos resultados para outras populações trans em diferentes contextos. O estudo utilizou dados secundários das fichas de atendimento, o que pode ter resultado em vieses devido à possível incompletude ou inconsistência nas informações registradas. Outra limitação é a perda de seguimento observada durante o acompanhamento clínico, especialmente após a segunda consulta. Tal perda pode ter influenciado os achados, uma vez que a análise considerou os três primeiros retornos de acompanhamento, o que limitou uma avaliação completa da continuidade do uso da PrEP a longo prazo.

Em conclusão, a maioria das pessoas trans que utilizam a PrEP em um serviço de saúde de referência no município de São Paulo era mulher trans, jovem, parda, com mais escolaridade. Alta descontinuidade no acompanhamento clínico após a segunda consulta foi observada e sugere necessidade de ações para aumentar a continuidade da PrEP. Considerando essa estreita janela de oportunidade, ações de prevenção estratégicas a essa população, como a vacinação contra hepatite B, deveriam ser priorizadas nas primeiras consultas.
